# Cutaneous toxicities from targeted therapies used in oncology: Literature review of clinical presentation and management

**DOI:** 10.1016/j.ijwd.2021.09.009

**Published:** 2021-09-28

**Authors:** Solène Huynh Dagher, Astrid Blom, Hedi Chabanol, Elisa Funck-Brentano

**Affiliations:** a Department of General and Oncologic Dermatology, Ambroise-Paré Hospital, Assistance Publique - Hôpitaux de Paris, Boulogne-Billancourt, France; b Research Unit EA 4340 “Biomarkers in Cancerology and Hemato-oncology,” University of Versailles-Saint-Quentin-en-Yvelines, Université Paris-Saclay, Boulogne-Billancourt, France; b Unité de Recherche Plaies et Cicatrisation, Institut Curie, Paris, France

**Keywords:** Molecular targeted therapies, antineoplastic agents, drug-related side effects and adverse events, dermatological toxicities, supportive care

## Abstract

With the development of molecular targeted therapies, a wide array of dermatologic toxicities is appearing. Their prevention, recognition, and management by dermatologists is critical to ensure antineoplastic treatment continuation. The objective of this study was to provide a literature review of the most common dermatologic toxicities due to targeted therapies in oncologic patients, including their clinical presentation, prevention, and management.

## Highlights

• Cutaneous toxicities are frequent with targeted therapies.
• Managing cutaneous toxicities is critical for life-saving treatment continuation.
• Dermatologists can provide a key input in preventing and managing these toxicities.

 

**What is known about this subject in regard to women and their families?**

• Adverse effects of targeted therapies reduce quality of life for patients undergoing cancer treatment.
• The families of patients undergoing targeted therapies treatment are involved in their care and seek to relieve these adverse effects.

**What is new from this article as messages for women and their families?**

• Some targeted therapies induce alopecia, trichomegaly, or hirsutism, with a strong esthetic and psychological impact on women.
• Poly-ADP ribose polymerase-inhibitors used in ovarian cancer have a specific cutaneous toxicity and photosensitivity that can be prevented with photoprotective measures.

## Introduction

Dermatologic toxicities are among the most frequently observed with targeted therapies. They are associated with a significant impact on quality of life (QoL) and treatment adherence. Indeed, studies report 75% to 90% of dermatologic adverse events (dAEs) among patients treated with targeted therapies (Rosen et al., 2013). dAEs result in lower QoL, including patient physical, emotional, and psychological well-being. In addition, dAEs jeopardize treatment adherence and optimal dose continuation and increase the risk of infection (Eilers et al., 2010). For example, dose interruptions and discontinuation are reported in 76% and 32%, respectively, of patients treated with epidermal growth factor receptor inhibitor (EGFRi) due to acneiform rash (Boone et al., 2007). Thus, the management of dAEs related to targeted therapies is a challenge for cancer outcomes.

Dermatologists can provide valuable assistance in preventing and managing dAEs, allowing the impact on QoL to be reduced and minimizing treatment dose reduction or discontinuation. Indeed, with the continued expansion of targeted therapies, dAEs are more and more diverse, some are drug-specific, and the expertise of dermatologists in preventing, recognizing, and managing these cutaneous toxicities is essential. Close collaboration between dermatologists and oncologists is critical to offer patients treated with targeted therapies optimal overall oncologic care.

The aim of this paper is to review the clinical presentation and management of dAEs in patients treated with targeted therapies in oncology.

### Targeted therapies

Targeted therapies can be inhibitors of the cellular membrane or intracellular molecular signaling pathways (Table 1). In the last 2 decades, the development of targeted therapies has revolutionized the prognosis of several cancers. The novel mechanisms of action through which these drugs achieve their effects have also resulted in the appearance of a new spectrum of adverse events. Given the pace of their development, dermatologists might not be familiar with their dermatologic toxicities. Knowledge of their mechanisms of action can be critical to understanding their toxicities. Targeted therapies inhibit signaling pathways in both malignant and normal cells, and epidermal and dermal homeostatic functions are particularly affected. Consequently, cutaneous, mucosal, hair, and nail toxicities are observed in most patients using these medications.

**Table 1:** Targeted therapies (nonexhaustive).

**Inhibitors of the cellular membrane**		
Epidermal growth factor receptor inhibitors	Monoclonal antibodies	Cetuximab, panitumumab, trastuzumab
	Specific tyrosine kinase inhibitors	Gefitinib, erlotinib
	Human epidermal growth factor receptor 2	Lapatinib
c-kit and breakpoint cluster region gene–Abelson proto-oncogene inhibitors		Imatinib, nilotinib, dasatinib, ponatinib
Angiogenesis agents	Vascular endothelial growth factor receptor inhibitors	Bevacizumab, ranibizumab
	Multikinase angiogenesis inhibitors	Vandetanib, pazopanib, sunitinib Pan-rapidly accelerated fibrosarcoma: Sorafenib, regorafenib
	Fibroblast growth factor receptor inhibitors	Infigratinib, erdafitinib, derazantinib, pemigatinib, futibatinib
**B. Inhibitors of intracellular molecular signaling pathways**		
Reticular activating system-RAF-MEK-ERK pathway	BRAF inhibitors	Vemurafenib, dabrafenib, encorafenib
	MEK inhibitors	Cobimetinib, trametinib, binemetinib
PI3K-protein kinase B-mTOR pathway	mTOR inhibitors	Everolimus, temsirolimus, rapamycin
	PI3K inhibitors	Idelalisib
Hedgehog signaling pathway	Hedgehog inhibitors	Vismodegib, sonidegib
JAK-signal transducer and activator of transcription pathway	JAK inhibitors	Ruxolitinib
PARP pathway	PARP inhibitors	Olaparib, rucaparib, niraparib

JAK = Janus kinase; MEK = mitogen-activated extracellular kinase; mTOR = mammalian target of rapamycin; PARP = poly ADP ribose polymerase; PI3K = phosphatidylinositol-3-kinase;

### Cutaneous toxicities, a predictive biomarker

Some of these drugs have specific dAEs, and their presence is associated with the efficacy of the drug. For example, the acneiform eruption induced by EGFRi has been reported to represent a surrogate marker of therapeutic response (Perez-Soler, 2006; Pérez-Soler et al., 2004; Wacker et al., 2007). Moreover, a meta-analysis of 12 cohort studies of patients treated with sorafenib (a multikinase angiogenesis inhibitor) reported a significant association between hand–foot skin reaction and reduced risk of death (hazard ratio: 0.45; *p* < .00001; Vincenzi et al., 2010). The association of other dAEs (xerosis, leukocytoclastic vasculitis, pruritus, and paronychia) with the efficacy of targeted therapies has been reported in previous studies (Rzepecki et al., 2018). However, more extensive prospective studies are needed to confirm these results.

## Dermatologic management: Generalities

Dermatologic toxicity by molecules and their management are summarized inTables 2 and 3.

**Table 2:** National Cancer Institute Common Terminology Criteria for Adverse Events, version 5

**Grade 1**	**Mild:** Asymptomatic or mild symptoms; clinical or diagnostic observations only; intervention not indicated
**Grade 2**	**Moderate:** Minimal, local, or noninvasive intervention indicated; limiting age-appropriate instrumental activities of daily life
**Grade 3**	**Severe** or medically significant, but not immediately life-threatening; hospitalization or prolongation of hospitalization indicated; disabling; limiting self-care activities of daily life
**Grade 4**	**Life-threatening** consequences; urgent intervention indicated
**Grade 5**	**Death** related to adverse event

**Table 3:** Cutaneous adverse events induced by targeted therapies: synthesis table.

	**EGFRi**	**c-kit inhibitors BCR**–**ABLi**	**Angiogenesis agents**	**FGFRi**	**BRAFi**	**MEKi**	**mTORi PI3Ki**	**HHi**	**JAKi**	**PARPi**
Papulopustular eruption	X					X				
Exanthema (nonspecific rash)		X			X	X				
Keratinocytic proliferation Eruption Benign and malignant neoplasms		X	X		X X		X		X	
Hand–foot skin reaction			X	X	X					
Hair changes (alopecia, trichomegaly, hirsutism, kinking)	X		X	X						
Nail changes Paronychia and pyogenic granuloma Onycholysis	X			X		X				
Xerosis/fissures	X			X		X				
Oral Mucositis Stomatitis Dysgeusia Xerostomia	X			X			X	X		
Hypopigmentation		X	X							
Photosensitivity reaction	X				X					X
Panniculitis					X					
Melanocytic lesion changes					X					

BCR–ABLi = breakpoint cluster region gene–Abelson proto-oncogene inhibitors; EGFRi = epidermal growth factor receptor inhibitors; FGFRi = fibroblast growth factor receptor inhibitors; HHi = hedgehog inhibitors; JAKi = Janus kinase inhibitos; MEKi = mitogen-activated extracellular kinase inhibitors; mTORi = mammalian target of rapamycin inhibitors; PARPi = poly-ADP ribose polymerase inhibitors; PI3Ki = phosphatidylinositol-3-kinase inhibitors

### Skin toxicity: General prevention measures

The management objective at this stage is to prevent dose reduction or discontinuation. The management principles must target skin inflammation, infection, and skin barrier defects, such as xerosis. Therefore, physicians should educate patients on preventive measures, such as avoiding alcohol-based lotions and irritating products, using gentle cleansers, regular use of emollients, and sun-protective measures (Bensadoun et al., 2013; Lacouture et al., 2021a).

The European Society of Medical Oncology clinical practice guidelines have been published recently and provide a useful recommendation for the management of dAEs related to targeted therapies (Lacouture et al., 2021a).

### Evaluation of dermatologic toxicity

#### Impact on quality of life

dAEs have a significant impact on health-related QoL (Joshi et al., 2010). Some studies showed discrepancies between physicians’ and patients' symptom evaluations. The use of patient-reported outcomes for symptom evaluation seems associated with clinical benefit (Basch et al., 2016; Fromme et al., 2004). Even if physicians grade dAEs as 1 or 2 per the Common Terminology Criteria for Adverse Events (CTCAE; Table 4), their chronicity and association with pain and pruritus result in a high impact on QoL. The negative impact from dAEs from targeted therapies is more significant than dAEs with cytotoxic agents (Rosen et al., 2013). EGFRi rash and pruritus produced the most significant negative impacts.

**Table 4:** Dermatologic toxicities of targeted therapies and management.

**Dermatologic toxicities**	**Most frequent agents**	**Management**	**Level of evidence**
Papulopustular eruption	EGFR and MEK inhibitors	**Prevention** Prophylactic therapy with oral tetracycline antibiotics for 6 to 8 weeks for patient with high risk	I, B
		**Treatment** Initiation or continuation of oral tetracycline antibiotics Low-/moderate-potency topical or oral corticosteroids Low-dose isotretinoin Culture-driven antibiotics if superinfection	I, B III IV, C I, B
Exanthema (nonspecific rash)	MEK, BRAF, and kinase inhibitors	**Treatment** Topical or oral corticosteroids and antihistamines	III
Paronychia and pyogenic granuloma	EGFR, MEK, mTOR inhibitors	**Prevention** Correction of lateral nail curvature, avoidance of repeated friction/trauma/excessive pressure, wearing gloves while cleaning Well-fitting shoes and cotton socks Antimicrobial soaks	IV, B
		**Treatment***Grade 1 and 2* Topical povidone-iodine 2%, topical antibiotics, high-potency local corticosteroids Topical beta-blockers *Grade 3 (or intolerable grade 2)* Cryotherapy Surgical treatment (partial nail avulsion) Culture-driven antibiotics, if needed	III, B III, B IV, B IV, B V
Keratinocytic proliferation			
Hand–foot skin reaction	Multikinase angiogenesis, BRAF and FGFR inhibitors	**Prevention** Limiting traumatic activities and the use of skin irritants Use of urea 10% cream Treatment of hyperkeratosis and orthopedic shoe	III I, B III
		**Treatment** Keratolytic agents and high-potency topical corticosteroids Lidocaine patches	III III
Skin neoplasms	BRAF (in monotherapy) and JAK inhibitors	**Warts and verrucal keratoses** Systemic retinoids in prevention Topical (keratolytics, 5-fluorouracil, imiquimod) or destructive measures **Squamous cell carcinomas and keratoacanthomas** Close dermatologic follow-up If few lesions, surgical excision If multiple, 5-fluorouracil, systemic retinoids, or photodynamic therapy	III III III III II, A/B
Hyperkeratotic rashes	BCR–ABL, pan-RAF, selective PI3K and angiogenesis inhibitors	Keratolytics, emollients, gentle skin care Low-/moderate-potency local corticosteroids	III III
Xerosis/fissures	EGFR, VEGFR, MEK and mTOR inhibitors	**Prevention** Limited shower time, gentle cleanser, alcohol-free lotions **Treatment** Emollients, ammonium lactate 12% cream, salicylic 6% cream (only on small surface areas) If eczematous reaction, moderate-potency topical corticosteroids For fissures, protective covering (hydrocolloid, biological glue, cyanoacrylate glue), barrier creams, and emollients	III IV III II, B
Hair changes			
Androgenic pattern alopecia	Hedgehog, FGFR, BRAF, and EGFR inhibitors	Topical minoxidil 5%	I, B
Inflammatory and scarring alopecia	Erlotinib	Topical steroids	III
Trichomegaly, hypertrichosis	EGFR inhibitors	Regular eyelash trimming Laser hair reduction	III I, B
Mucosal changes			
Stomatitis	mTOR and FGFR inhibitors	**Prevention** Education on oral cavity hygiene, dental work Avoidance of salty, spicy, citrus-based food and hot beverages **Treatment** Potent topical corticosteroids, antiseptic washes, and local anesthetics Local lubricants Ophthalmologic consultation to avoid ocular complications	IV IV IV V V
Mucositis (oral, genital, and ocular sphere)	EGFR inhibitors		
Hypopigmentation	c-kit, BCR–ABL, and multikinase angiogenesis inhibitors	Reversible after treatment discontinuation	
Photosensitivity reaction	Vemurafenib (BRAF inhibitor), EGFR inhibitors, PARP inhibitors	**Prevention** Strict sun protection **Treatment** Wet dressings, emollients, topical corticosteroids Short course of systemic corticosteroids or NSAID	II, B III III
Panniculitis	BRAF inhibitors	Short course of systemic corticosteroids or NSAID	III
Melanocytic lesion changes	BRAF inhibitors	Close dermatologic follow-up with dermoscopic examination and photographs	II, B

BCR–ABL = breakpoint cluster region gene–Abelson proto-oncogene; EGFR = epidermal growth factor receptor; FGFR = fibroblast growth factor receptor; JAK = Janus kinase; MEK = mitogen-activated extracellular kinase; mTOR = mammalian target of rapamycin; NSAID = nonsteroidal antiinflammatory drug; PARP = poly-ADP ribose polymerase; PI3K = phosphatidylinositol-3-kinase; VEGFR = vascular endothelial growth factor receptor

In the absence of evidence-based recommendations, these recommendations respond to an expert consensus based on data from the literature and personal experiences. Levels of evidence are defined according to different categories based on types of studies (Shekelle et al., 1999): IA) Evidence from meta-analysis of randomized controlled trials; IB) evidence from at least one randomized controlled trial; IIA) evidence from at least one controlled study without randomization; IIB) evidence from at least one other type of experimental study; III) evidence from nonexperimental descriptive studies, such as comparative studies, correlation studies, and case-control studies; IV) case series (and poor-quality cohort and case-control study); and V) evidence from expert opinions or clinical experience of respected authorities, or both.

Thus, it is advisable to use patient-reported tools to measure the severity and the impact on QoL. General dermatology tools are available (e.g., Skindex-16, Skindex-29, Dermatologic Life Quality Index, and DIELH-24), as well as symptom-specific tools (e.g., Functional Assessment Of Cancer Therapy-Epidermal Growth Factor Receptor Inhibitors-18 and Hand–Foot Syndrome 14; Chan et al., 2015).

The management of toxicity is well codified, using the CTCAE, version 5, grading scale (Table 4). A scale is provided for each type of dAE, which helps standardize treatment and toxicity management.

### General management and alert signs

When dAEs reach grade 3 or 4 (or grade 2 if perceived as intolerable by the patient), treatment should be interrupted until symptoms are reduced to grade 0 or 1. A rechallenge at a lower dose is then recommended. No treatment interruption is recommended for grade 1 or 2 dAE, unless specified in the package insert. Dose modification should be performed as recommended in the manufacturer's package.

Targeted therapies are rarely responsible for severe or life-threatening drug reactions, such as Steven–Johnson syndrome/toxic epidermal necrolysis, drug reaction with eosinophilia and systemic symptoms, or acute generalized exanthematous pustulosis. In patients exhibiting a maculopapular rash, signs of a more serious reaction can include a diffuse, dark, purpuric, and painful exanthem; facial edema; the presence of vesicles, bullae, or pustules; mucosal damage; fever; adenopathy; or laboratory findings of neutrophilia, hypereosinophilia, and elevated liver enzyme levels. Prompt recognition and appropriate management are required.

Many targeted therapies may be related to a nonspecific maculopapular rash or morbilliform eruption with mild symptoms. All classes can be involved, although kinase inhibitors, BRAF inhibitors (BRAFi), and MEK inhibitors (MEKi) are more commonly responsible for morbilliform eruptions. Patients can be managed with topical or oral corticosteroids and antihistamines (Cury-Martins et al., 2020). Regardless of the specific dAEs of targeted therapies, these can, like all drugs, be the cause of severe drug eruptions. The prevention and treatment of each toxicity are detailed in Table 3, as well as the levels of evidence.

### Dermatologic adverse events

#### Papulopustular exanthema

**Figure 1:**
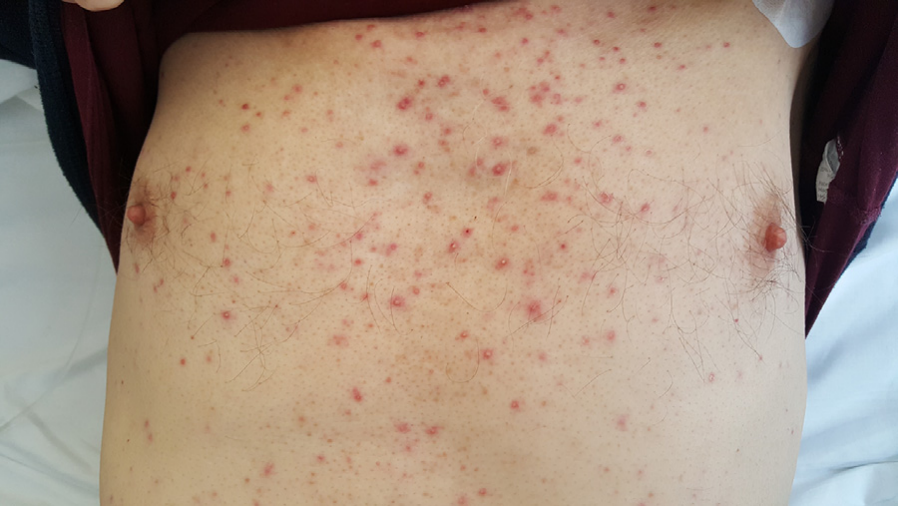
Papulo-pustular eruption with EGFR inhibitors

Papulopustular exanthema is characterized by an eruption of papules and pustules in a seborrheic distribution (face, chest and upper back; Fig. 1). Although commonly described as an acneiform rash, this reaction lacks comedones and nodulocysts and is often itchy. A papulopustular exanthem is the most common EGFRi dAE, but it is also reported with MEKi. More than 75% of patients treated with EGFRi experience these eruptions, with 10% to 20% reaching grades 3 to 4 (Lacouture, 2006). Onset after treatment initiation is 1 to 2 weeks (Macdonald et al., 2015), and the mean duration is 9.4 weeks (Braden and Anadkat, 2016). Although CTCAE grading is typically low, the impact on patients’ QoL is often disproportionately high due to symptoms of burning, stinging, and tenderness.

#### Complications

Bacterial superinfection (most often caused by *Staphylococcus aureus*) occurs in 23% to 29% of patients (Table 3; Braden and Anadkat, 2016). When suspected (pustules on the arms, legs, and trunk, as well as yellow crust and discharge), cultures should be obtained not only for bacteria, but also viruses and fungi, and appropriate therapies administered orally or intravenously.

#### Prevention

For selected patients with an identified higher risk (history of severe acneiform rash, radiotherapy, or chemotherapy in association with EGFRi, or no dermatologic follow-up), oral tetracyclines (doxycycline 100 mg twice a day or minocycline 100 mg once daily) may be prescribed prophylactically for 6 to 8 weeks to prevent complications (Table 3; Jatoi et al., 2008; Lacouture et al., 2021a; Scope et al., 2007).

#### Treatment

For grade 1/2 rash, initiation or continuation of oral tetracycline antibiotics and topical corticosteroids for at least 6 weeks is recommended (Table 3; Lacouture et al., 2021a). The mean treatment duration to control the eruption is 3 months (Park et al., 2021). For a grade 3 rash, in addition to the previous measures, a short course of systemic corticosteroids is suggested, with an interruption of the targeted therapy until the rash resolves to grade 0/1. Low-dose isotretinoin can also be used, but only after tetracyclines have been discontinued to lower the risk of cerebral edema.

### Paronychia/periungual pyogenic granuloma

**Figure 2:**
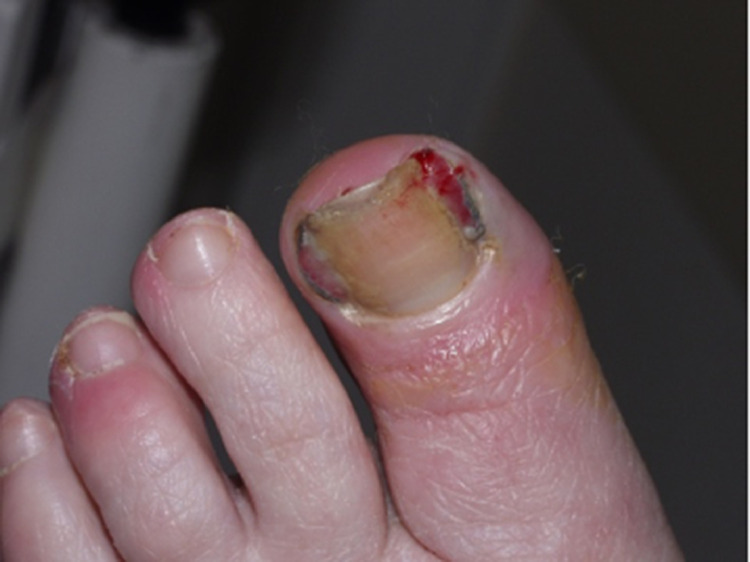
Paronychia and pyogena granuloma like lesion with MEK inhibitors (melanoma)

Nail toxicities, such as paronychia and pyogenic granuloma-like lesions, are well-recognized dAEs of EGFRi, MEKi, and mammalian target of rapamycin inhibitors (mTORi). They are distinct from chemotherapy-induced lesions, usually observed on the nail plate or nail matrix, and can significantly impair a patient's QoL. Paronychia occurs in 17.2% of patients treated with EGFRi (Garden et al., 2012) and, to a lesser extent, with MEKi and mTORi (Robert et al.. 2015). Lesions mostly affect the toenails and thumbs and develop several weeks or months after treatment onset. Paronychia starts with the development of periungual inflammation and can evolve into pyogenic granuloma-like lesions (i.e., lesions with friable granulation tissue on the lateral and/or proximal nail folds, mimicking ingrown nails; Fig. 2). Nail bed and matricial changes, such as onycholysis, brittle nails, and slower nail growth rate, are less frequent (Lacouture and Sibaud, 2018).

#### Prevention

Patient education with preventive measures, such as preventive correction of lateral nail curvature with a podiatrist, avoidance of cutting nails too short or biting nails, avoidance of repeated friction/trauma/excessive pressure, wearing gloves while cleaning, the use of antimicrobial soaks, and wearing comfortable and well-fitting shoes and cotton socks. Patients should be closely monitored for early signs of inflammation (Table 3; Lacouture et al., 2021a; Robert et al., 2015).

#### Treatment

Numerous therapeutic options are available, with variable rates of success. For grades 1 and 2, local treatments include topical povidone-iodine 2%, topical antibiotics, and high-potency corticosteroids. A recent study reported the efficacy of topical beta-blockers (timolol 0.5% gel twice daily, under occlusion for 30 days) in nine patients (Table 3; Sollena et al., 2019). For grade 3 (or intolerable grade 2), surgical treatment (nail plate avulsion with physical destruction of excessive granulation tissue) is indicated. Cryotherapy allowed complete resolution of the granuloma lesions in all patients in a prospective study including 135 patients after a mean of 1.58 (range, 1-4) treatments (Mirshams et al., 2006). If a secondary infection is suspected, bacterial/viral/fungal cultures should be obtained and proper antibiotics prescribed.

## Hyperkeratotic skin reaction

Hyperkeratotic skin adverse events are characterized by the disruption of epidermal homeostasis and interaction with keratinocytic proliferation or differentiation caused by targeted therapies. Their clinical presentation varies.

### Hand–foot skin reaction

**Figure 3:**
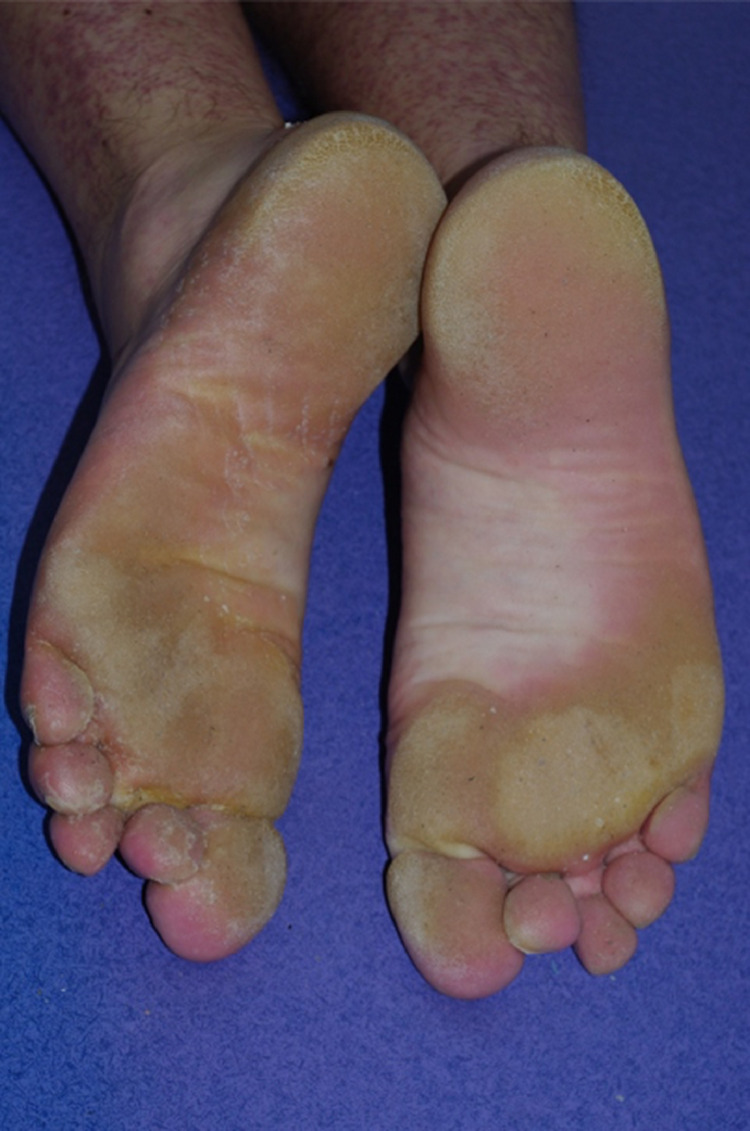
Hand-Foot skin reaction with BRAF inhitors (melanoma)

A hand–foot skin reaction induced by targeted therapies differs from that of chemotherapies. It appears as more localized, symmetrical, yellowish, hyperkeratotic lesions with erythematous margins at pressure-bearing areas on the palms and soles (Fig. 3), which are generally painful. A localized hand–foot skin reaction emerges 1 to 5 weeks after multikinase angiogenesis inhibitor initiation in 5% to 60% of patients (Vastarella et al., 2020). Female sex is an independent risk factor for the development of the condition (Chanprapaph et al., 2016; Dranitsaris et al., 2012). The reason, in the case of dose-dependent toxicities such as hand–foot skin reaction, may be higher doses relative to body weight or body surface area in women. BRAFi and fibroblast growth factor receptor inhibitor (FGFRi) can also cause similar lesions, albeit less frequently, especially with the association of MEKi with BRAFi (Lacouture and Sibaud, 2018; Lacouture et al., 2021b).

Prevention includes the use of well-fitting shoes, limiting activities that are traumatic for the feet (e.g., long walks) that might worsen the hyperkeratosis due to friction, avoidance of skin irritants, and the use of urea 10% cream three times per day (Table 3). If prominent hyperkeratosis is detected before treatment initiation, we recommend a podiatrist consultation to treat calluses and the use of an orthopedic shoe insert if needed (Lacouture et al., 2021a).

Hyperkeratosis is treated with keratolytic agents (salicylic acid 5%-10% or urea 10%-40%) and skin inflammation with high-potency topical corticosteroids (Table 3; Lacouture et al., 2021a). Lidocaine 5% patches can be used for analgesia. Given its dose-dependent nature, a dosage adjustment of the targeted therapy can be performed.

### Epidermal neoplasms and hyperkeratotic rashes

Epidermal neoplasms include benign lesions (warts, verrucal keratoses) and malignant lesions (keratoacanthomas, squamous cell carcinomas [SCCs]), as well as inflammation of actinic/seborrheic keratoses. These dAEs have been observed with BRAFi in monotherapy in 10% to 30% of patients (Belum et al., 2015). However, combination with MEKi significantly reduces their development (Carlos et al., 2015; Russo et al., 2017; Sanlorenzo et al., 2014). Several cases of eruptive or aggressive SCCs have been reported with Janus kinase inhibitors as well (Aboul-Fettouh and Nijhawan, 2018; Fabiano et al., 2015).

Recently, hyperkeratotic rashes, such as keratosis pilaris-like rash, pityriasis rubra pilaris-like rash, Grover's disease, and induced psoriasis, have been described with the use of a new generation. The molecules responsible are the new generation breakpoint cluster region gene-Abelson protooncogene inhibitors, pan-RAF inhibitors, selective phosphatidylinositol-3-kinase inhibitors, or angiogenesis inhibitors (Vastarella et al., 2020).

In patients treated with BRAFi in monotherapy, close dermatologic follow-up is necessary to detect SCCs as early as possible (Table 3). The combination of BRAFi with MEKi is the rule to improve survival and reduce dAEs. Retinoids may be considered for patients with extensive verrucal keratoses or multiple SCCs (Anforth et al., 2013).

Topical treatments (keratolytics, 5-fluorouracil, or imiquimod; Table 3) can be used for benign neoplasms. SCCs should be resected surgically with appropriate margins or, in case of multiple lesions, 5-fluorouracil, systemic retinoids, or photodynamic therapy should be preferred. Disruption of anticancer therapies is not usually necessary.

### Xerosis and skin fissures

**Figure 4:**
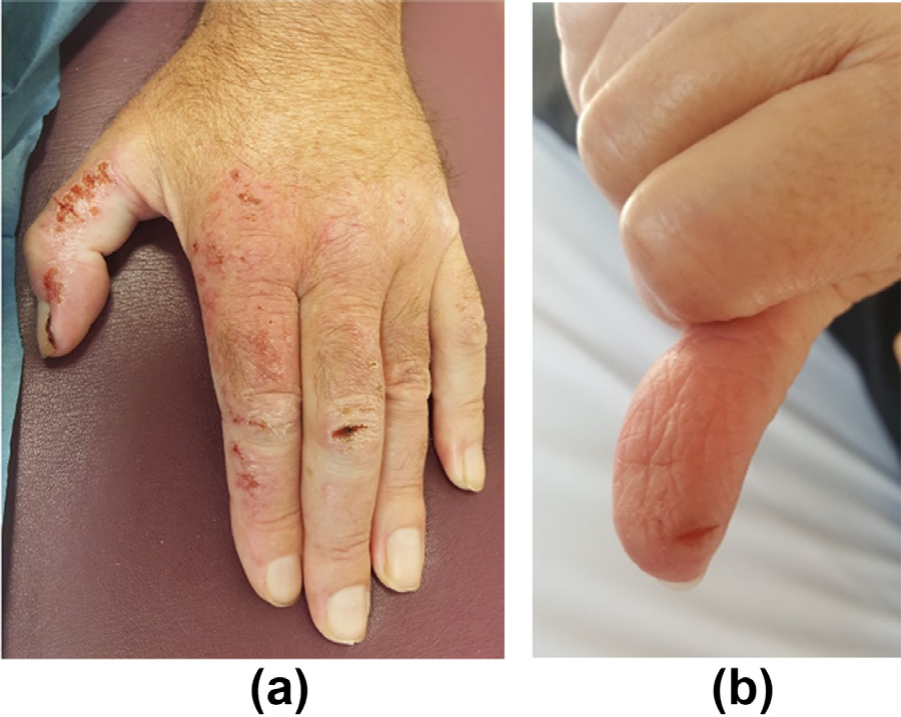
Xerosis (A) and fissures (B) with EGFR inhibitors

Fifteen percent to 20% of patients receiving targeted therapies develop xerosis. EGFRi, vascular endothelial growth factor receptor inhibitors, MEKi, and mTORi are associated with the highest rates (Valentine et al., 2015). For example, xerosis develops in 47% of patients on panitumumab (an EGFRi). Skin fissures and deep cracks localized in the fingertips, palms, or knuckles can form due to significant xerosis (Figs. 4A and B).

### Prevention and treatment

To prevent xerosis, short tepid showers are preferable to immersion in a tub. Gentle cleansers (pH neutral soaps or syndets) and alcohol-free moisturizers should also be preferred (Table 3). Treatment consists of barrier creams, such as occlusive moisturizing creams, and ammonium lactate 12% cream or salicylic acid 6% cream for scaly areas (only on small surface areas to prevent salicylism; Table 3; Lacouture et al., 2011; Valentine et al., 2015). Topical corticosteroids should be used on eczematous areas. For fissures, protective coverings, such as hydrocolloid, biological glue, or even cyanoacrylate glue, can rapidly relieve pain. Thick emollients (e.g., petroleum jelly and zinc oxide ointment) can be used to promote healing.

### Hair changes

Although reversible, alopecia is cited as the most disturbing anticipated adverse event in up to 58% of women's cancer treatment. Moreover, women experiencing alopecia report lower self-esteem, poorer body image, and lower QoL. A reversible, androgenic pattern of alopecia, usually of mild-to-moderate severity (grades 1-2) develops with a number of targeted therapeutics. For example, it occurs in 63% of patients treated with Hedgehog inhibitors (Sekulic et al., 2012), 26% to 46% of those treated with FGFRi (Lacouture et al., 2021b), 14% to 19% with BRAFi (Carlos et al., 2015), and more rarely with EGFRi. Erlotinib-induced cicatricial alopecia and inflammatory, nonscarring alopecia has been described as well (Pongpudpunth et al., 2009; Yang et al., 2011).

**Figure 5:**
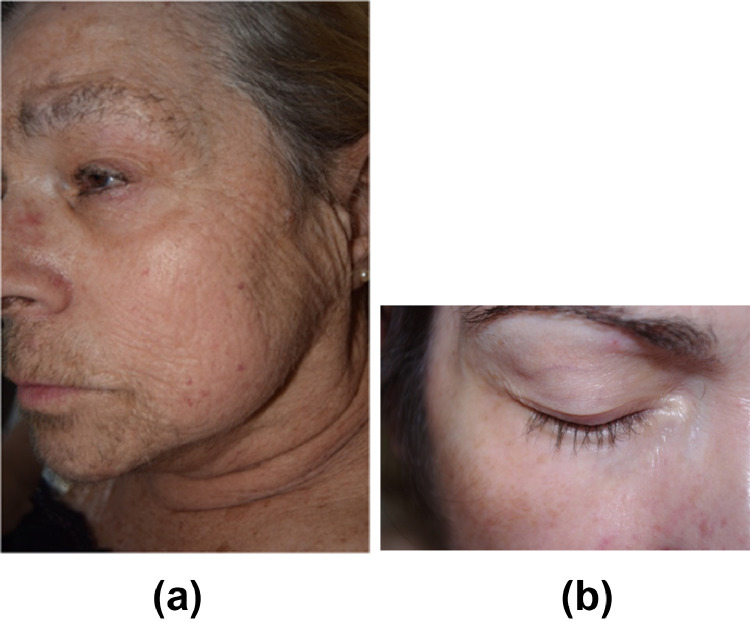
Hypertrichosis with cetuximab and afatinib (EGFR-mutated bronchial adenocarcinoma) (A) and trichomegaly with MEK inhibitors (melanoma) (B)

Modification in hair growth, texture, and quality can be seen starting on the second or third month of treatment with EGFRi (Macdonald et al., 2015); the hair adopts a fine and brittle quality and becomes kinky. Trichomegaly and hirsutism of the upper lip in women have been described with EGFRi. Reversible hair depigmentation has been described with multikinase angiogenesis inhibitors (cabozantinib; Figs. 5A and B; Zuo et al., 2015).

### Prevention and treatment

No methods to prevent hair dAEs from targeted therapies are known. Topical minoxidil 5% may stimulate hair growth in patients exhibiting an androgenic pattern of hair loss, and topical corticosteroids can be used for inflammatory and scarring hair loss (Table 3; Macdonald et al., 2015). Trichomegaly can be treated with regular eyelash trimming to avoid keratitis, and hypertrichosis can be treated with laser hair reduction. Depilatory creams are not recommended because they can disrupt the skin barrier, which is already compromised in most patients. Hair camouflaging methods may also be considered. In the absence of scarring alopecia, hair changes are generally reversible.

### Oral mucositis and stomatitis

Stomatitis is the most common adverse event related to mTORi, with an overall incidence in 32% to 57% of patients (Gomez-Fernandez et al., 2012). It can be severe and result in dose adjustments and interruption in 16% of patients (Atkins et al., 2004). Stomatitis is also frequently observed in patients treated with FGFRi (up to 65% with erdafitinib), where it is characterized by painful, well-defined lesions. Dry mouth or xerostomia are also commonly reported with FGFRi.

Mucositis involving oral (aphthae, xerostomia, geographic tongue) and, to a lesser extent, genital (vulvovaginitis, balanitis, and genital aphthae) and ocular (conjunctivitis and keratitis) mucosa are noted with EGFRi (Lacouture, 2006). Dysgeusia may develop with hedgehog inhibitors, because this pathway is involved in taste perception. For example, >50% of patients treated with the hedgehog inhibitor vismodegib developed grade 1 or 2 dysgeusia (Sekulic et al., 2012).

#### Prevention

Dental disease should be eliminated before treatment initiation through corrective work, and patients should be taught good oral cavity hygiene. Patients should avoid foods that injure or irritate the mucosa, such as those that are salty, spicy, or citrus-based, as well as hot beverages (Table 3; Lacouture et al. 2021b).

#### Treatment

Stomatitis and oral mucositis grade 1 or 2 can be treated with potent topical corticosteroids (Chuang and Langone, 2007), antiseptic washes, or local anesthetics (Macdonald et al., 2015). Local lubricants ameliorate discomfort from vaginal and ocular dryness. Ophthalmologic consultation should be considered to avoid ocular complications (Table 3; Melichar and Němcová, 2007).

### Pigmentary changes

Hypopigmentation of the skin (diffuse or localized) and hair is commonly reported during treatment with c-kit inhibitors, BCR-ABL inhibitors, and multikinase angiogenesis inhibitors. The physiopathology is explained by the role of c-kit in regulating melanogenesis, proliferation, migration, and survival (Besmer et al., 1993). The depigmentation is reversible when treatment is stopped. No prevention or treatment is available.

### Photosensitivity reaction

When considering vemurafenib (a BRAFi) plus cobimetinib (a MEKi), a specific adverse event is represented by ultraviolet A-induced photosensitivity (related to the drug's chemical structure), occurring in 48% of patients, with 5% presenting with grade ≥3 (Ascierto et al., 2016). Vemurafenib in monotherapy can cause similar lesions, albeit less frequently (Russo et al., 2017). Photosensitive rashes were also reported with EGFRi, supporting in vitro data showing that EGFRi results in altered keratinocyte survival in response to ultraviolet radiation. Photosensitivity reactions have also been reported with poly-ADP ribose polymerase inhibitors (17%, any grade), which is used in particular to treat ovarian cancers (LaFargue et al., 2019).

#### Prevention

Prevention measures rely on strict sun precautions, including photoprotective clothing and the use of broad-spectrum sunscreens (including ultraviolet A; Table 3). Ultraviolet light–blocking window films can be applied to the car and home windows for added protection.

#### Treatment

Topical treatments, such as wet dressings, emollients, and corticosteroids, are useful (Table 3). A short course of systemic corticosteroids or nonsteroidal antiinflammatory drugs can be initiated.

### Other cutaneous adverse events specific to particular agents

Some less frequent dAEs from targeted therapies are specific to BRAFi. Modification of pigmented lesions, such as eruptive melanocytic nevi, changes in existing nevi, and even melanoma, have been reported, primarily in the first 5 months after treatment initiation (Göppner et al., 2014; Perier-Muzet et al., 2014). Careful skin examination, including dermoscopy and the use of photographs to document changes in pigmented lesions and appearance of new ones, is required during BRAFi treatment. Panniculitis localized on the upper and lower extremities usually resolves without treatment interruption and is managed with a short course of corticosteroids or nonsteroidal anti-inflammatory drugs. Nevertheless, dose adjustment or discontinuation may be necessary if the pain is too severe.

Selective FGFRi give rise to a new specific spectrum of dAEs, including alopecia, stomatitis, xerosis, and hand–foot skin reaction. Onycholysis is almost always evident, and supportive care with nail avulsion and dose adjustment may be necessary (Bétrian et al., 2017). Calcinosis cutis/calciphylaxis has been observed in two patients treated with FGFRi (infigratinib and pemigatinib; Lacouture et al., 2021b).

### Particular impacts on women

The female sex is an independent risk factor in some specific dAEs due to targeted therapies. In patients treated with sorafenib for advanced clear-cell carcinoma, the relative risk of hand–foot skin reactions increases by 68% for women (Dranitsaris et al., 2012). Other studies showed that women were predisposed to sorafenib-induced high-grade skin rashes (Tsuchiya et al., 2013) or even to sorafenib-induced erythema multiform (Ikeda et al., 2012). High-grade skin rashes were also found to be associated with the dose per body weight and body surface area (Tsuchiya et al., 2013). Consequently, a lower body weight or body surface area in women may explain the higher incidence of sorafenib-induced skin toxicities.

Among targeted therapies with dAEs, drugs that are more likely to be used for cancers specific to women are HER2 inhibitors (lapatinib), which is indicated for the treatment of metastatic breast cancer with overexpression of HER2 receptors, and poly-ADP ribose polymerase inhibitors (olaparib, rucaparib, niraparib), which are indicated in the treatment of ovarian cancer.

## Conclusion

Dermatologists have a crucial role in collaborating with oncologists to identify and manage the frequent dAEs related to targeted therapies. Like all types of adverse effects induced by anticancer treatments, dAEs are best managed by a combination of CTCAE grading and a QoL measure, using specific, validated tools. Interventions aimed at prevention and appropriate early treatments can allow patients to receive anticancer treatment at the right dose and duration, which is fundamental for disease-specific survival.

## Study approval

N/A

## Conflicts of interest

None.
